# Seroprevalence and Risk Factors of *Toxoplasma gondii* Infection among Pregnant Women in Kumasi: A Cross-Sectional Study at a District-Level Hospital, Ghana

**DOI:** 10.1155/2021/6670219

**Published:** 2021-04-02

**Authors:** Bhavana Singh, Linda Batsa Debrah, Godfred Acheampong, Alexander Yaw Debrah

**Affiliations:** ^1^Department of Clinical Microbiology, School of Medicine and Dentistry, Kwame Nkrumah University of Science and Technology, Kumasi, Ghana; ^2^University Hospital, Kwame Nkrumah University of Science and Technology, Kumasi, Ghana; ^3^Kumasi Centre for Collaborative Research in Tropical Medicine, Kwame Nkrumah University of Science and Technology, Kumasi, Ghana; ^4^Faculty of Allied Health Sciences, Kwame Nkrumah University of Science and Technology, Kumasi, Ghana

## Abstract

**Background:**

This study investigated the prevalence and risk factors of *Toxoplasma gondii* infection among pregnant women in a district-level hospital in Ghana and compared the diagnostic performance of the rapid diagnostic test (RDT) and enzyme-linked immunosorbent assay (ELISA) for *T*. *gondii* diagnosis.

**Method:**

This cross-sectional study included 400 consecutive consenting women in their first-trimester stage of pregnancy. A validated well-structured closed-ended questionnaire was used to collect sociodemographic data and possible risk factors of each participant. Blood samples were collected for analysis of *T*. *gondii* IgG and IgM using the commercial ELISA Kit and RDT.

**Results:**

Seroprevalence of toxoplasmosis was 21.5% and 57.3% based on the RDT and ELISA technique, respectively. Secondary education (cOR = 1.9, 95% CI (1.1-3.1), and *p* = 0.020) and contact with cats (cOR = 1.7, 95% CI (1.1-2.8), and *p* = 0.030) were significant predictors of *T*. *gondii* infection, with the former being the only independent risk factor for *T*. *gondii* infection (aOR = 1.8, 95% CI (1.0-3.0), and *p* = 0.034) by the ELISA method. The sensitivity, specificity, and area under the curve (AUC) of RDT-IgM against ELISA were 42.9%, 95.9%, and 0.694, respectively, whereas those of RDT-IgG were 31.0%, 91.2%, and 0.611, respectively. The diagnostic consistency between the two methods was fair for both RDT-IgM (*κ* = 0.304) and RDT-IgG (*κ* = 0.201).

**Conclusion:**

The prevalence of *T*. *gondii* infection among pregnant women at Kumasi is 21.5% and 57.3% based on the RDT and ELISA technique, respectively. Secondary education and contact with cats were the major risk factors of *T*. *gondii* infection. Using ELISA as the reference, the RDT used in this study for the diagnosis of *T*. *gondii* infection has low sensitivity, and therefore, it is unreliable. However, this finding does not invalidate all RDTs because there are several other brands of RDT with good sensitivity and specificity. Further studies to ascertain the performance of other commercially available RDT kits are needed.

## 1. Introduction

Toxoplasmosis is a disease caused by an obligate intracellular protozoan parasite, *Toxoplasma gondii* (*T*. *gondii*). It is widely distributed and can affect humans, pets, and livestock [1]. In a general population, *T*. *gondii* infection can remain asymptomatic but can also cause lymphadenopathy and flu-like symptoms, which may lead to eye disease, most frequently chorioretinitis [2]. The parasite in its inactive state remains in an individual without presentable signs but flares up upon immunosuppression [3, 4].

Pregnant women constitute a specific risk group, and primary infection may be acquired during pregnancy that may lead to abortion, stillbirth, and neurological disorders in the unborn child [5, 6]. Evidence from the literature indicates that the prevalence of *T*. *gondii* infection among pregnant women ranges from less than 1% to 92% depending on the geographical location [2, 4, 7]. Among African populations, studies in Tanzania [8] and Ghana [9] reported *T*. *gondii* seroprevalence of 30.9% and 92.5%, respectively, among pregnant women, and factors such as eating undercooked or cured meat, having a cat as a pet, low educational level, contact with soil, crowded conditions, parity, and consumption of raw vegetables were noted as predisposing factors [8, 9].

More so, a higher prevalence is observed in tropical countries with a humid and warm climate, and conversely, a lower prevalence is found in colder countries. Several anthropogenic factors explain a large part of the variations in human seroprevalence such as dietary habits in terms of the method of cooking meat, kinds of meat or vegetables consumed, and vegetable cleaning; handwashing techniques adopted by a community; economic, social, or cultural habits; quality of water; and sanitation coverage [10–12]. Also, seroprevalence increases with age and the rate of acquiring infection in relation to age varies according to the country and socioeconomic level. Seroprevalence in children living under poor hygienic conditions is high, probably linked to telluric or waterborne contamination by oocyst ingestion. This implies that water is an important source of human infection in areas where humans use unfiltered surface water for consumption [10–12].

The burden of *T*. *gondii* infection is reported to be generally severe in immunocompromised populations such as pregnant women where studies have linked this condition to severe pregnancy outcomes [5, 13]. Infection with *T*. *gondii* in early pregnancy often leads to severe consequences such as abortion and abnormalities, as compared to the asymptomatic presentation of the newborn when infected during the third trimester [14].

Due to the detrimental effects of *T*. *gondii* infection on the mother and her unborn baby, early diagnosis of the infection is key. There are several laboratory tests for the diagnosis of *T*. *gondii*, including the rapid diagnostic test (RDT), serological tests (such as enzyme-linked immunosorbent assay (ELISA)), PCR, and histological and cytological examination of tissue and body fluids [15]. RDT is easy to perform, is rapid, and does not need trained personnel and special equipment, and therefore, it is suitable for convenient testing [16]. For this reason, most diagnostic services in some African countries such as Ghana rely on RDT. However, most RDT kits on the market have not been field-evaluated. Moreover, the unreliability of RDT for *T*. *gondii* diagnosis has been reported [14]. Thus, other relatively less expensive tests like ELISA are being advocated. One advantage of ELISA is its ability to detect specific anti-*Toxoplasma* IgG and IgM antibodies with higher accuracy compared to RDT, although skilled personnel is required [16].

This study is thus aimed at assessing the prevalence and risk factors of *T*. *gondii* infection among pregnant women attending antenatal care at the Kwame Nkrumah University of Science and Technology (KNUST) Hospital in Ghana and comparing the diagnostic performance of the RDT and ELISA technique for *T*. *gondii* diagnosis.

## 2. Materials and Methods

### 2.1. Study Design/Setting

This cross-sectional study, which was part of a larger longitudinal study, was conducted in Kumasi Metropolis at the Kwame Nkrumah University of Science and Technology (KNUST) Hospital from August 2017 to February 2018. The KNUST Hospital caters to a population of over 200,000 per annum, and it is made of 21,000 students, 30,000 staff and dependents, and about 150,000 people from over 30 surrounding communities. The University Health Services offers services in general medical care as well as specialist services.

### 2.2. Sample Size

The sample size for the study was calculated using Fischer's sampling formula (*N* = *Z*^2^*PQ*/*d*^2^), where *Z* is the critical value of the normal distribution (1.96 at 95% CI); *P* is the estimated prevalence of *T*. *gondii* among pregnant women in Ghana (51.2%) [17]; *Q* = 100 − *P*; and *d* is the absolute precision or sampling error tolerated = 5%. From the above equation, the minimum sample size for this study was 384. However, in an effort to increase the statistical power of the study, we recruited 400 consecutive consenting pregnant women attending antenatal care at the KNUST Hospital.

### 2.3. Ethical Approval

Ethical approval was obtained from the Committee on Human Research Publication and Ethics (CHRPE) of the School of Medicine and Dentistry of the Kwame Nkrumah University of Science and Technology, Kumasi (CHRPE/AP/541/17). Informed consent, either signed or thumbed, was obtained from every participant before enrolling them in this study.

### 2.4. Questionnaire Administration

A validated questionnaire, designed by reviewing previous studies of similar objectives, was used to collect information on sociodemographic factors such as age, marital status, level of education, income, behavioral practices including eating and handling of meat, contact with cats and level of handling cats or cat litter, and obstetric history in terms of parity which is the number of times a woman has given birth.

### 2.5. Sampling Procedure

Two millimeters (2 ml) of venous blood was drawn from each participant. The blood samples were transported to the Kumasi Centre for Collaborative Research (KCCR) laboratory in an ice chest. Plasma was prepared from the whole blood sample by spinning it at 1500 rpm for 10 min, and aliquots were stored at -20°C until subsequent analysis.

### 2.6. Sample Analysis

Each sample was tested for the presence of *Toxoplasma* antibodies using both the rapid diagnostic test (RDT) IgG and IgM kits (Innovation Biotech TOXO IgG/IgM RDT, Beijing, China) and the commercial ELISA Kit (INVBIO IgG and IgM ELISA, Innovation Biotech Beijing Co. Ltd., China) following the manufacturer's instructions. *T*. *gondii* antigen detection was performed by the sandwich ELISA method (INVBIO IgG and IgM ELISA, Innovation Biotech Beijing Co. Ltd., China) according to the manufacturer's instructions. Briefly, all reagents were allowed to attain room temperature before use. The wash buffers were diluted at a 1 : 40 ratio of distilled water before use. One hundred microliters (100 *μ*l) of the sample dilution liquid was added in each well, and 10 *μ*l of the sample was added. The resultant solution was mixed thoroughly, covered with an adhesive cover, and incubated at 37°C for 20 min. After incubation, the mixture was aspirated from the wells followed by four washes with the wash solution. Residual wash solution droplets were removed by blotting the microtitre plate onto an absorbent paper. A 50 *μ*l conjugate was added and incubated at 37°C for 20 minutes, and a 100 *μ*l of tetramethylbenzidine (TMB) solution (prepared from 50 *μ*l of chromogen A and 50 *μ*l of chromogen B) was pipetted into each well, mixed gently, and incubated at 37°C for 10 min. Fifty microliters (50 *μ*l) of Stop Solution was added to each well and gently mixed for 30 s to stop the reaction. The absorbance of each well was measured spectrophotometrically at 450 nm using a Thermo Electron Multiskan EX plate reader (Shanghai, China).

### 2.7. Data Analysis

Statistical analysis was done using a statistical package SPSS (version 25). To compare the diagnostic consistency between RDT and ELISA, Cohen's kappa coefficient (*κ*) was used. Diagnostic accuracy tests including sensitivity, specificity, positive predictive value (PPV), negative predictive value (NPV), and area under the curve (AUC) were performed using ELISA as the reference.

The associations between *T*. *gondii* infection and possible risk factors were tested with a chi-squared test. The magnitude of associations was assessed using odds ratios (OR) at a 95% confidence interval (CI). A multivariate logistic regression model was used to identify the explanatory variables among confounding risk variables that would explain the occurrence of toxoplasmosis. A *p* value < 0.05 was considered statistically significant.

## 3. Results

### 3.1. Demographic Characteristics of the Study Participants

Approximately 51.0% of the study participants were 30-39 years old. A higher proportion of the participants (37.5%) had no prior children; 41.8% had low-income salaries (<$100 per month); 47.8% had a family size of 1-3; 30% were unskilled workers; and 39.8% had completed tertiary education. The majority (86.6%) of the study subjects were married (Table 1).

A higher percentage of the study participants used pipe-borne water (67.0%) and water closest for their organic waste disposal (67.3%). In terms of cooking and eating practices, a major percentage (84.3%) of the participants ingested raw vegetables, about 36.2% of the study participants eat raw meat without cooking, 95.0% used clean cooking surfaces and utensils after contact with risk materials, 86.5% handled meat, and 42.5% have constant contact with soil. In terms of pet handling practices among seropositive participants, 36.7% owned pets and 20.5% owned cats. About 27.5% had contact with cats, and 83.4% did not have a litter box for cats. Among subjects that had a litter box for cats, a major proportion (85.7%) did not use gloves in handling it and 67.9% did not practice handwashing after contact with cats (Table 2).

### 3.2. *Toxoplasma gondii* Seroprevalence and Associated Factors

**Figure 1:**
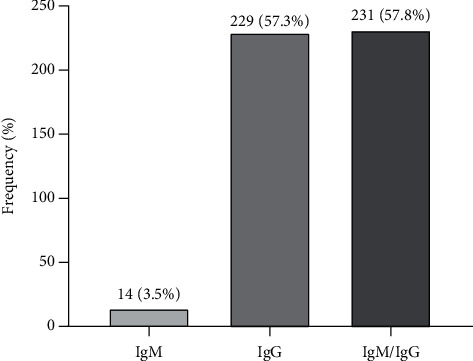
Seroprevalence of *Toxoplasma gondii* antibodies among the study participants by ELISA. Percentages were calculated from the number of positives over the total number of participants.

Overall, the seroprevalence of IgM, IgG, and IgM/IgG *T*. *gondii* antibodies by ELISA was 3.5%, 57.3%, and 57.8%, respectively (Figure 1).

**Figure 2:**
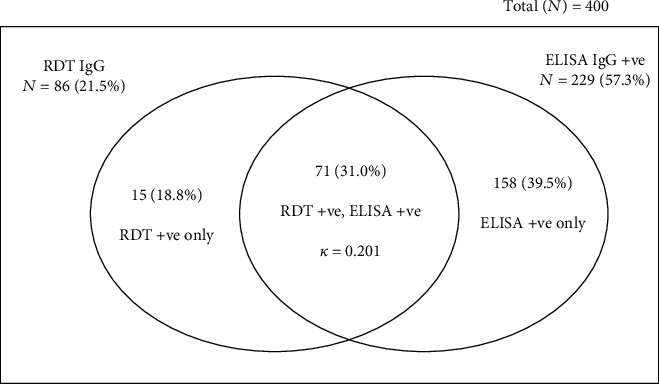
Seroprevalence of *Toxoplasma gondii* antibodies (IgG) among the study participants. The number of IgG-positive cases based on RDT was compared with ELISA using kappa statistics.

**Figure 3:**
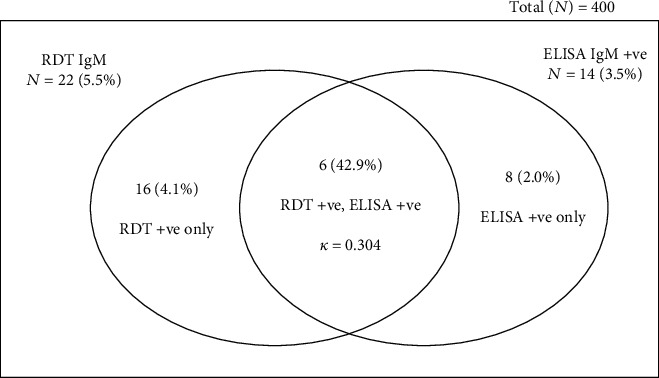
Seroprevalence of *Toxoplasma gondii* antibodies (IgM) among the study participants. The number of IgM-positive cases based on RDT was compared with ELISA using kappa statistics.

Out of the 400 pregnant women screened for *T*. *gondii* IgG, 86 (21.5%) were positive with RDT and 229 (57.3%) were positive for ELISA. Upon stratification by the antibody type, the percentage of the participants detected by RDT-IgG only as positives was 8.8% and that by ELISA only was 39.5% (Figure 2). Also, the percentage of IgM positives detected by RDT alone was 4.1% while that by ELISA was 2.0% (Figure 3).

Secondary education (cOR = 1.9, 95% CI (1.1-3.1), and *p* = 0.020) and contact with cats (cOR = 1.7, 95% CI (1.1-2.8), and *p* = 0.030) were significant predictors of *T*. *gondii* infection, with the former being the only independent risk factor for *T*. *gondii* infection (aOR = 1.8, 95% CI (1.0-3.0), and *p* = 0.034) by the ELISA method (Table 3).

### 3.3. Validation of the Rapid Diagnostic Test Using the ELISA Technique

**Figure 4:**
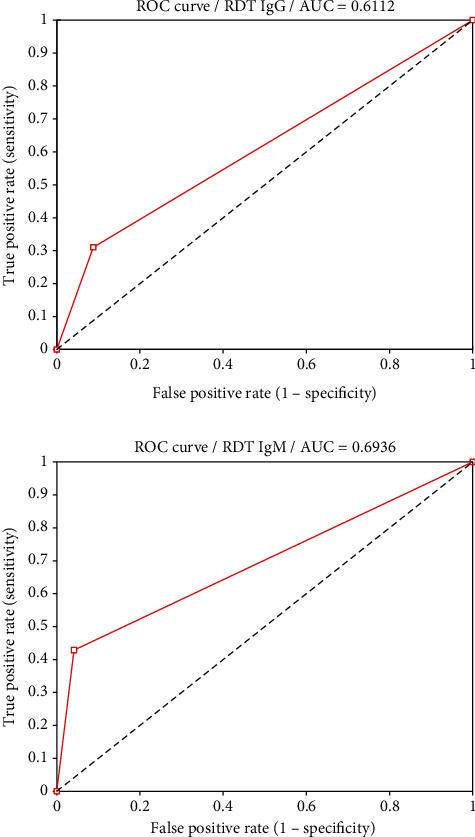
Receiver operating characteristic (ROC) curve showing the diagnostic consistency.

The sensitivity and specificity of RDT-IgG using ELISA as the gold standard were 36% and 91.2%. There was a fair diagnostic agreement (kappa = 0.201) between the RDT-IgG and ELISA IgG tests. The sensitivity and specificity of RDT-IgM were 43% and 96% with a fair diagnostic agreement (kappa = 0.304). The effectiveness of RDT-IgM (AUC = 0.694) compared with RDT-IgG (AUC = 0.611) was better using ELISA as the gold standard (Figure 4 and Table 4).

## 4. Discussion

This study investigated the prevalence of *T*. *gondii* infection among pregnant women in Kumasi Metropolis using the commercially available rapid diagnostic test (RDT) and ELISA test kits that detect anti-*Toxoplasma* IgG and IgM antibodies. Infection with *T*. *gondii* in early pregnancy presents the risk of fetal transmission, where the rates of transmission range between 60% and 81% in the third trimester [15, 16]. Classically, congenital infection results from primary acquired maternal infection during gestation. The severity of fetal infection is inversely correlated to the stage at which infection occurs, and 80% of neonates are asymptomatic when infected during the third trimester of gestation [14]. However, in the first-trimester transplacental transmission, the consequences for fetal development are heavy and severe, often leading to severe abnormalities or to abortion. Neonatal manifestations of congenital toxoplasmosis have been related to fetal conditions, including hydrocephalus, microcephaly, intracranial calcifications, retinochoroiditis, strabismus, blindness, epilepsy, and psychomotor and mental retardation [17–19]. Hence, a timely method of detection of *T*. *gondii* infection in pregnant women is key.

The result showed that more than half of the study participants had an overall IgG- and IgM-positive infection (57.8%), indicative of acute and previous exposure. *T*. *gondii* IgG seropositivity only was 57.3% in our study, indicative of past or previous exposure. Even though the seroprevalence was high in this study, it was relatively low as compared to the 95.9% detected by Ayi et al. [9] in Accra, in southern Ghana. In our study, *T*. *gondii* IgM positivity was found in only 3.5% of our participants and all other seropositive cases were IgG, indicating chronic infection. Several other similar studies have reported no or few IgM positivity compared to IgG [18]. In line with our *T*. *gondii* IgM seropositivity, a systematic review by Bigna et al. [36] indicated a global IgM seroprevalence of 1.9%, with the highest being in Eastern Mediterranean (4.1%) and America being the lowest in *T*. *gondii* IgM (1.1%) seroprevalence. Moreover, global IgG seroprevalence was found to be 32.9% which is lower than our findings. In Srilanka, however, Iddawela et al. [37] found a *T*. *gondii*-specific IgM seropositivity as low as 0.3% and IgG seropositivity far lower than our finding, that is, 29.9%, indicating that almost 70.1% of their population are susceptible to primary acute infection during pregnancy and possible fetal anomalies. In patients with acute infection, *T*. *gondii*-specific IgM appears initially and antibody levels become negative in few months. However, persistent IgM positivity in chronic stages of *Toxoplasma* infection has been reported [19]. Thus, the presence of IgM antibodies does not always confirm acute infection. However, the negative *Toxoplasma* IgM test rules out recently acquired *Toxoplasma* infection [20]. In antibody detection for diagnosis of toxoplasmosis, anti-IgM suggests recent, acute, or ongoing infections and IgM antibodies indicate acute infections because these antibodies are not usually in acquired immunity and are very rare in chronic infections [9, 21].

This study is the first of its kind in Kumasi Metropolis, and the high seroprevalence is not surprising as human infection is widely reported, with nearly one-third of the world population being exposed to the parasite [22]. As the location is an urban area and not a rural one, toxoplasmosis is not exclusive to marginalized communities. In high-income countries, like the USA and the UK, it has been estimated that between 10% and 40% of the population is infected, while in Central and South America and continental Europe, the prevalence ranges from 50% to 80% [23]. In our study, IgM antibodies were detected in 3.5% of the study population, implying the possible presence of acute infection.

Importantly, in this study, there was an association between contact with cat and seropositivity of *Toxoplasma*, consistent with reports by previous studies [9, 13]. Cats have been implicated in the transmission of *T*. *gondii* infection. Owning cats and subsequent exposure to their feces facilitate the transmission of the parasite [9]. Congruently, Al Hamdani and Mahdi [13] also found that *Toxoplasma* antibodies were more prevalent in pregnant women with cats at home than in pregnant women that did not own cats. Iddawela et al. [37] in Srilanka, on the other hand, did not find the association between *T*. *gondii* seropositivity and owning a cat, but rather preparation and selling raw meat (*p* = 0.05) and household gardening (*p* = 0.01) were significantly associated with being seropositive with *T*. *gondii*.

We also found low educational status to be associated with higher *T*. *gondii* seroprevalence, which is consistent with a study by Kwakye-Nuako in Ghana [24] who also found a higher prevalence of *T*. *gondii* infection among secondary school graduates and participants with no education as compared to university graduates. Furthermore, being married was also associated with a higher seroprevalence *T*. *gondii* infection and this is in line with a study by Singh et al. [25]. Frimpong et al. [39] also found that being a farmer and being involved in construction work showed a 15.5 times likelihood of contracting the infection in Lusaka, as well as the socioeconomic status of the pregnant women that had an inverse relationship with infection. There is an extent of diversity when it comes to the level of education and occupation, as different studies investigate different economic activities under this term and others do not specify exactly which activities were investigated.

Eating uncooked meat and handling of meat were not significantly associated with toxoplasmosis in our study, and it is in line with Frimpong et al. [39] in Lusaka. However, several other studies have reported handling of raw meat and ingestion of undercooked meat to be associated with toxoplasmosis [37, 40]. The definition of uncooked meat may differ among these studies due to different settings, such as culture and preference, under which these studies were carried out. This study considered uncooked meat to be meat not adequately cooked for more than 30 minutes. This is, however, not a documented universal standard since undercooked meat in some European countries refers to raw meat which still has blood in it, and this is uncommon in our setting. However, the criteria other studies used to define undercooked meat in their studies are unclear, which could explain why this variable did not come out as an important determining factor of the infection in this study.

In comparing the diagnostic utility of RDT and ELISA, RDT-IgM performed better in diagnosing *T*. *gondii* infection than RDT-IgG. This finding is in harmony with a study by Bassiony et al. [26]. Contrary to our results, Huang et al. [27] reported a high RDT sensitivity and specificity of 97.2 and 95.8%, respectively, in the detection of anti-*T*. *gondii* antibodies in the cat's sera. The relative agreement was 96.1% between RDT and ELISA. The disparity between our findings and that of Huang et al. could be due to the differences in the type of material tested (human sample versus cat sample). It is important to note that despite the advantage of the RDT test of being rapid and easy to perform and requiring no special expertise or high technological machines to adjudge positive or negative results, a higher concentration and purity of the antigen are required for this test [28], and sensitivity is most often challenged. Notwithstanding the low sensitivity recorded by the RDT used in this study, there are several other commercial RDTs that have demonstrated higher sensitivities, and therefore, their importance and efficiency for mass screening cannot be completely overlooked considering the convenience and cost benefits of RDT in resource-poor countries [37].

Overall, this study has shown a moderate to high seroprevalence of *T*. *gondii* infection among pregnant women in Kumasi. However, these results cannot be generalized to the whole country as it focused on females attending the ANC at the KNUST Hospital, mostly a semiurban educated population. Levels of exposure to toxoplasmosis could however vary in more rural areas where involvement in agricultural activities and soil contact is greater. Therefore, more studies need to be done in other areas in a larger population with sociodemographic variation to represent the whole country. Also of note, only one brand of RDT was utilized in this study and the sensitivity/specificity may vary when a different brand is used. We recommend that future studies take other brands of RDT used in the country into consideration. Furthermore, the few studies conducted in Ghana showed moderate to high seroprevalence in the population [9, 29]. Moreover, since congenital toxoplasmosis can only occur in pregnant women primarily infected with *T*. *gondii* during pregnancy, the serological status at the beginning of pregnancy is very important. Women who are seropositive in their first trimester have a limited risk of giving birth to babies with congenital toxoplasmosis, and hence, preventive measures can help reduce the risk of infection during pregnancy in seronegative women. There is therefore the need to follow up the seroconversion of seronegative mothers for the next two trimesters to identify women with recent *Toxoplasma* infection and to detect the possible transmission rate to the babies born from the seroconverted mothers.

## 5. Conclusion

The prevalence of *T*. *gondii* infection among pregnant women at Kumasi is 21.5% and 57.3% based on the RDT and ELISA technique, respectively. Secondary education and contact with cats were the major risk factors of *T*. *gondii* infection. Using ELISA as the gold standard, our brand of RDT for the diagnosis of *T*. *gondii* infection is less reliable. However, this finding does not invalidate all *Toxoplasma* RDTs because there are several other brands of RDT with good sensitivity and specificity. Further studies to ascertain the performance of other commercially available RDT kits are needed.

We recommend *Toxoplasma* screening at antenatal clinics in Ghana. Since *T*. *gondii* infection is acquired predominantly from the environment, health education of the pregnant women will be an inexpensive but significant mode of reducing the risk of acquisition and transmission of the infection.

## Figures and Tables

**Table 1:** Sociodemographic factors and *Toxoplasma gondii* seroprevalence.

Variable	*T*. *gondii* seroprevalence		*χ* ^2^, df	*p* value
	Negative (171) *N* (%)	Positive (229) *N* (%)		
Age (years)			0.97, 2	0.616
20-29	73 (42.7)	109 (47.6)		
30-39	91 (53.2)	112 (48.9)		
40 and above	7 (4.1)	8 (3.5)		
Parity			1.02, 4	0.907
0	70 (40.9)	88 (38.4)		
1	34 (20.5)	56 (24.9)		
2	41 (24.7)	53 (23.5)		
3	18 (10.8)	24 (10.6)		
>3	7 (4.2)	9 (4.0)		
Marital status			7.95, 2	*0*.*019*
Married	156 (91.2)	191 (83.4)		
Single	11 (6.4)	35 (15.3)		
Separated	4 (2.3)	3 (1.3)		
Educational level			9.27, 4	*0*.*049*
Primary	9 (5.3)	18 (7.9)		
Junior high	39 (22.8)	59 (25.8)		
Senior high	35 (20.5)	61 (26.6)		
Tertiary	82 (48.0)	77 (33.6)		
No formal education	6 (3.5)	14 (6.1)		
Income (*N* = 269)			1.13, 2	0.588
Low income (<$100 per month)	66 (58.4)	101 (64.7)		
Middle income ($100-400 per month)	46 (40.7)	54 (34.6)		
High income (>$400 per month)	1 (0.9)	1 (0.6)		
Occupation			4.17, 4	0.384
Unskilled	44 (25.7)	76 (33.2)		
Skilled	33 (19.3)	39 (17.0)		
Self-employed	35 (20.5)	40 (17.5)		
Professional	38 (22.2)	40 (17.5)		
Unemployed	21 (12.3)	34 (14.8)		
Family size (*N* = 361)			2.08, 2	0.353
1-3	89 (56.7)	102 (50.0)		
4-6	64 (40.8)	93 (45.6)		
>6	4 (2.5)	9 (4.4)		

**Table 2:** Hygienic predisposing factors and *Toxoplasma gondii* seroprevalence.

Variable	*T*. *gondii* seroprevalence		*χ* ^2^, df	*p* value
	Negative *N* (%)	Positive *N* (%)		
Source of water			1.22, 1	0.285
Pipe-borne	109 (63.7)	158 (69.0)		
Borehole	62 (36.3)	71 (31.0)		
Waste disposal facility			3.11, 2	0.211
WC	123 (74.5)	145 (66.2)		
Public toilets	41 (24.8)	72 (32.9)		
Land, rivers, and streams	1 (0.6)	2 (0.9)		
Ingestion of raw vegetables			1.19, 1	0.332
No	23 (13.5)	40 (17.50)		
Yes	148 (86.5)	189 (82.5)		
Ingestion of uncooked meat			0.02, 1	0.916
No	61 (35.7)	80 (34.9)		
Yes	110 (64.3)	149 (65.1)		
Cleaning cooking materials			1.40, 1	0.258
No	6 (3.5)	14 (6.1)		
Yes	165 (96.5)	215 (93.9)		
Meat handling (e.g., beef mutton)			0.38, 1	0.558
No	21 (12.3)	33 (14.4)		
Yes	150 (87.7)	196 (85.6)		
Contact with soil			1.35, 1	0.262
No	104 (60.8)	126 (55.0)		
Yes	67 (39.2)	103 (45.0)		
Owning pets			1.13, 1	0.339
No	117 (68.4)	145 (63.3)		
Yes	54 (31.6)	84 (36.7)		
Owning cats			3.39, 1	0.083
No	148 (86.5)	182 (79.5)		
Yes	23 (13.5)	47 (20.5)		
Contact with cats			4.79, 1	*0*.*027*
No	140 (81.9)	166 (72.5)		
Yes	31 (18.1)	63 (27.5)		
Housing of cats			1.11, 1	0.304
No	143 (83.6)	182 (79.5)		
Yes	28 (16.4)	47 (20.5)		
Litter box for cats			2.99, 1	0.109
No	153 (89.5)	191 (83.4)		
Yes	18 (10.5)	38 (16.6)		

**Table 3:** Bivariate and multivariate analyses of risk factors significantly associated with *Toxoplasma gondii* seroprevalence in this present study.

Risk factors	Number tested	% positives	Univariate		Multivariate	
			OR (95% CI)	*p* value	OR (95% CI)	*p* value
Marital status						
Married	347	55.0	1.6 (0.4-7.4)	0.525	1.4 (0.3-6.6)	0.640
Single	46	76.1	4.2 (0.8-21.9)	0.085	3.4 (0.6-18.1)	0.149
Separated	7	42.9	1 (reference)		1 (reference)	
Educational level						
No formal education	20	70.0	2.5 (0.9-6.8)	0.076	2.6 (0.9-7.1)	0.066
Primary	27	66.7	2.1 (0.9-5.0)	0.084	2.0 (0.8-4.7)	0.121
Junior high	98	60.2	1.6 (1.0-2.7)	0.067	1.5 (0.9-2.6)	0.107
Senior high	96	63.5	1.9 (1.1-3.1)	0.020	1.8 (1.0-3.0)	0.034
Tertiary	156	48.4	1 (reference)		1 (reference)	
Owning cats						
No	330	55.2	1 (reference)		1 (reference)	
Yes	70	67.1	1.7 (1.0-2.9)	0.067	1.3 (0.6-2.6)	0.517
Contact with cats						
No	306	54.2	1 (reference)		1 (reference)	
Yes	94	67.0	1.7 (1.1-2.8)	0.030	1.3 (0.7-2.4)	0.475

**Table 4:** Diagnostic performance of *T*. *gondii* RDT-IgG and RDT-IgM diagnostic tests against the ELISA technique.

Diagnostic test	Sensitivity	Specificity	Accuracy	PPV	NPV	Cohen's kappa	AUC
RDT-IgG	0.31	0.912	0.56	0.38	0.83	0.201	0.611
RDT-IgM	0.43	0.960	0.94	0.27	0.97	0.304	0.694

NPV: negative predictive value; PPV: positive predictive value; AUC: area under the curve.

## Data Availability

The datasets used and/or analyzed during the current study are available from the corresponding author on reasonable request.
